# Branching paths: Unveiling functional divergence of PIF1 and PIF4 via promoter and protein variations

**DOI:** 10.1093/plcell/koae152

**Published:** 2024-05-22

**Authors:** Arpita Yadav

**Affiliations:** Assistant Features Editor, The Plant Cell, American Society of Plant Biologists; Biology Department, Penn State University, University Park, PA 16802, USA

Plants use complex photoreceptors, including phytochromes, to detect and respond to light. Phytochromes detect red and far-red light, triggering light-dependent activities from seed germination to blooming. Phytochromes fluctuate between active (Pfr) and inactive (Pr) forms in response to red and far-red wavelengths, affecting gene expression and physiological reactions. Phytochrome interacting factors (PIFs) are basic helix loop helix (bHLH) transcription factors that play important roles in light signaling, growth, and development. They act as cellular signaling hubs, integrating external and internal signals in plants (Krahmer and Fankhauser 2023). PIFs regulate gene expression by binding to the promoter DNA of target genes. They play different functions in seedling growth, germination, and temperature response. The Arabidopsis genome encodes 8 PIFs (PIF1-8) based on the presence of an Active Phytochrome B binding (APB) motif; however, PIF1 and PIF3 also possess an Active Phytochrome A binding motif in addition to the APB (Khanna et al. 2004).

PIF genes are believed to have originated early in the evolution of plants and underwent diversification through both whole-genome duplication and local gene duplication events (Jiang et al. 2022). The functional diversification of PIF genes encompasses the promoter as well as the protein coding region. In this issue, **Hanim Kim, Nayoung Lee, and coauthors (Kim et al. 2024)** provide insights into the influence of promoters and protein coding regions in shaping PIF functions. This study focusses on PIF1 and PIF4, 2 functionally different PIF genes, to fill this gap. The authors exchange promoters and analyze chimeric proteins to determine how promoters (influencing *mRNA* level) and the protein itself (protein function and stability) affect PIF gene functional diversity.

*PIF1* regulates seed germination, whereas *PIF4* influences hypocotyl elongation in response to red light and high ambient temperature. For *PIF1*, the promoter effect was found to play a more substantial role than that of the protein (Fig.). For *PIF4*, protein function plays a larger role in red light, with the promoter effect and protein function having equal importance at high ambient temperature (Fig. 1). Additional experiments revealed that the amino-terminal regions of the PIF1 and PIF4 proteins are pivotal in their functional diversification. Distinct patterns within these areas can engage with various proteins, impacting the regulation of gene expression and durability. For example, the HOOKLESS1 transcription factor, an ethylene signaling component, was found to interact more strongly with PIF4 than PIF1, in accord with the major role of PIF4 in the regulation of hypocotyl elongation. The *PIF1* and *PIF4* promoters possess unique cis-regulatory elements, which contribute to their divergent mRNA expression patterns. The expression of *PIF4* is controlled by both the circadian clock and temperature through unique binding sites for clock components and temperature-responsive transcription factors. Conversely, there is a smaller number of regulators that are recognized for their influence on *PIF1* expression.

**Figure 1.**
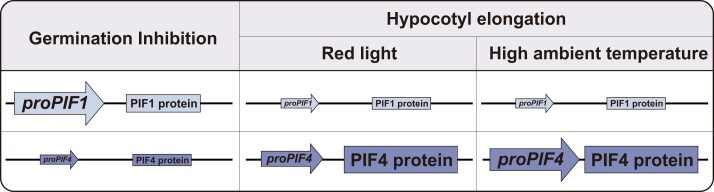
Diversification of *PIF1* and *PIF4* functions. The *PIF1* promoter predominantly controls the activity of *PIF1* in regulating seed germination, with a lesser influence from the PIF1 protein. For PIF4, the protein-coding sequence plays a larger role in the activity of the *PIF4* gene in regulating hypocotyl elongation in red light, with a lesser contribution from the *PIF4* promoter. Both the *PIF4* promoter and PIF4 protein are necessary for the *PIF4* gene to stimulate the elongation of the hypocotyl at high ambient temperature. Arrows indicate promoters (*proPIF1* and *proPIF4*). Squares represent the PIF1 and PIF4 proteins. The relative sizes of the arrows and squares indicate the relative importance of the promoter and the protein in the functional diversification of PIF1 and PIF4 genes in each light response. Reprinted from Kim et al. (2024), Figure 7.

Ultimately, the functional diversification of *PIF1* and *PIF4* arises from an intricate interaction between the promoters and protein-coding regions, with different levels of influence depending on the individual light responses (Fig. 1). Gaining knowledge about these pathways offers a valuable understanding of how plants adjust to environmental stimuli and undergo developmental processes.

## Dive Curated Terms

The following phenotypic, genotypic, and functional terms are of significance to the work described in this paper:

PIF1 Gramene: AT2G20180
PIF1 Araport: AT2G20180

PIF4 Gramene: AT2G43010
PIF4 Araport: AT2G43010

PIF3 Gramene: AT1G09530
PIF3 Araport: AT1G09530
